# pH and ROS sequentially responsive podophyllotoxin prodrug micelles with surface charge-switchable and self-amplification drug release for combating multidrug resistance cancer

**DOI:** 10.1080/10717544.2021.1905750

**Published:** 2021-04-05

**Authors:** Chao Li, Yifan Wang, Shuo Zhang, Jiaojiao Zhang, Fang Wang, Yunhao Sun, Lirong Huang, Wen Bian

**Affiliations:** a Department of Infectious Disease, Wuhu No. 1 People’s Hospital, Wuhu, China; b Department of Oncology, Yancheng No. 1 People’s Hospital, Yancheng First Hospital Affiliated Hospital of Nanjing University Medical School, Yancheng, China; c Department of Cardiothoracic Surgery, Yancheng No. 1 People’s Hospital, Yancheng First Hospital Affiliated Hospital of Nanjing University Medical School, Yancheng, China

**Keywords:** Multidrug resistance, ROS-sensitive, pH-responsive, polymeric prodrug, charge reverse

## Abstract

Multidrug resistance (MDR) is one of the main reasons for tumor chemotherapy failure. Podophyllotoxin (PPT) has been reported that can suppress MDR cancer cell growth; however, effective delivery of PPT to MDR cancer cells is challenged by cascaded bio-barriers. To effectively deliver PPT to MDR cancer cells, a PPT polymeric prodrug micelle (PCDMA) with the charge-conversion capability and self-acceleration drug release function are fabricated, which is composed of a pH and reactive oxygen species (ROS) sequentially responsive PPT-polymeric prodrug and an ROS generation agent, cucurbitacin B (CuB). After reach to tumor tissue, the surface charge of PCDMA could rapidly reverse to positive in the tumor extracellular environment to promote cellular uptake. Subsequently, the PCDMA could be degraded to release PPT and CuB in response to an intracellular high ROS condition. The released CuB is competent for generating ROS, which in turn accelerates the release of PPT and CuB. Eventually, the released PPT could kill MDR cancer cells. The *in vitro* and *in vivo* studies demonstrated that PCDMA was effectively internalized by cancer cells and produces massive ROS intracellular, rapid release drug, and effectively overcame MDR compared with the control cells, due to the tumor-specific weakly acidic and ROS-rich environment. Our results suggest that the pH/ROS dual-responsive PCDMA micelles with surface charge-reversal and self-amplifying ROS-response drug release provide an excellent platform for potential MDR cancer treatment.

## 1. Introduction

Chemotherapy remains a fundamental approach to cancer treatment (Chen et al., 2016; Zhou et al., 2017). However, the development of multidrug resistance (MDR) by cancer cells during treatment is a major factor responsible for chemotherapy failure and accounts for over 90% of patient deaths (Zhou et al., 2018). Despite the complexity of MDR, over-expression of P-glycoprotein (P-gp) has been recognized as a major mechanism, which causes a reduction in the accumulation of drugs in cancer cells by increasing ‘active efflux’ and decreasing influx, thereby preventing tumor cells from being killed by drugs (Persidis, 1999; Gottesman et al., 2002; Joshi et al., 2017). To date, various strategies have been developed to effectively overcome the MDR of cancer cells, which mainly rely on the combination of chemotherapeutic drugs and MDR inhibitors (Sivak et al., 2017). However, the toxicity of P-gp inhibitors and difficulty in accurately regulating combination ration has greatly limited the efficacy of combination strategies (Li et al., 2019; Niu et al., 2019). Thereby, specific chemotherapeutic agents that can effectively suppress cancer cell growth and are not a substrate of P-gp may effectively help to overcome MDR.

Podophyllotoxin (PPT), a toxic lignan polyphenol extracted from the roots of the genus *Podophyllum*, has a high activity to fight lung, breast, leukemia, and other cancers (Kang et al., 2014; Chen et al., 2017). PPT is an anti-tubulin drug that can bind to tubulin to inhibit the formation of mitotic spindles during cell division (Ou et al., 2019). Recently, it has been demonstrated that PPT can effectively overcome P-gp-mediated MDR (Roy et al., 2015; Feng et al., 2020). However, the extremely low water solubility and side effects (e.g. gastrointestinal disorders and bone marrow suppression) have greatly hindered the clinical application of PPT (Roy et al., 2015; Zhou et al., 2018; Ou et al., 2019). Thereby, a rationally designed delivery system to effectively deliver PPT to MDR cancer cells may efficiently overcome MDR and strengthen the clinical application of PPT.

Recently, stimuli-responsive polymeric prodrug micellar-based drug delivery systems (PPM-DDS), designed by conjugating chemotherapeutics drugs to polymers through stimuli-sensitive linkages, have emerged as a promising platform for cancer therapy (Zhou et al., 2018; Hu et al., 2020; Yang et al., 2020). PPM-DDS combined the benefit of prodrug strategy (e.g. avoid premature drug release during blood circulation) and polymer micelles (e.g. good biocompatibility) can effectively surmount the barriers of the conventional chemotherapeutic agent (e.g. poor solubility) and enhance antitumor efficacy (Peer et al., 2007; Wang et al., 2019; Xu et al., 2020). Although PPM-DDS has achieved encouraging results in preclinical studies, only a few PPM-DDS have been put into clinical trials (Yin et al., 2019). Several issues still impeded the clinical translation of PPM-DDS, especially poor cancer cellular uptake, and intracellular incomplete drug release (Taresco et al., 2018; Dai et al., 2019; Wang et al., 2019).

To abundantly accumulate in tumor tissue via enhanced permeability and retention (EPR) effect, camouflaging PPM-DDS with stealth coronas is an effective strategy to offer sufficient blood circulation time and eventually accumulate in tumor tissue (Liu et al., 2020). However, PPM-DDS with a negative charge is difficult to be entered into cancer cells, whereas the positively charged ones have a higher affinity to cell membranes (Xu et al., 2018; Oddone et al., 2019; Kim et al., 2020; Wang et al., 2020). However, positively charged PPM-DDS can strongly bind to serum components, which will rapidly clear from the blood by the reticuloendothelial system (RES) (Lv et al., 2014; Pan et al., 2020). Fortunately, charge-conversion PPM-DDS triggered by tumor tissue acidic pH value (pH ≈ 6.8) has great potential to overcome this problem (Yang et al., 2017, 2018; Li et al., 2019). The charge-converting PPM-DDS could maintain a negative charge under the physiological environment (pH 7.4) to reduce nonspecific interactions with serum components and to avoid clearance by the RES, while the charge could be switched to positive on exposure to weakly acidic tumor tissue pH to enhance tumor uptake (Feng et al., 2016; Lim et al., 2017; Tang et al., 2017).

Moreover, after being internalized by cancer cells, the PPM-DDS should possess a complete drug release property that is also tumor-specific. Previous studies have shown that the concentration of reactive oxygen species (ROS), such as superoxides (O_2_^–^) and hydrogen peroxides (H_2_O_2_), is increased in cancer cells (Xu et al., 2017; Tao & He, 2018; Oddone et al., 2019; Pan et al., 2020; Tian et al., 2021; Zhou et al., 2021). The concentration of H_2_O_2_ in normal cells is relatively low at approximately 0.001–0.7 μM, while in cancer cells its concentration can reach up to 10–100 μM (Tao & He, 2018). Hence, PPM-DDS with an ROS-responsive strategy is much more tumor-specific and thus holds particular promise for enhancing the exposure of cancer cells to therapeutic agents (Hu et al., 2017). Nevertheless, tumor heterogeneity may affect the ROS-mediated drug release progress, where the endogenous ROS concentration insufficiently triggered the complete drug release (Ye et al., 2017). Thereby, pH/ROS-responsive charge-switchable PPM-DDS with ROS production capability may be an effective strategy for increased drug release selectivity and tumor treatment efficacy.

**Scheme 1.**
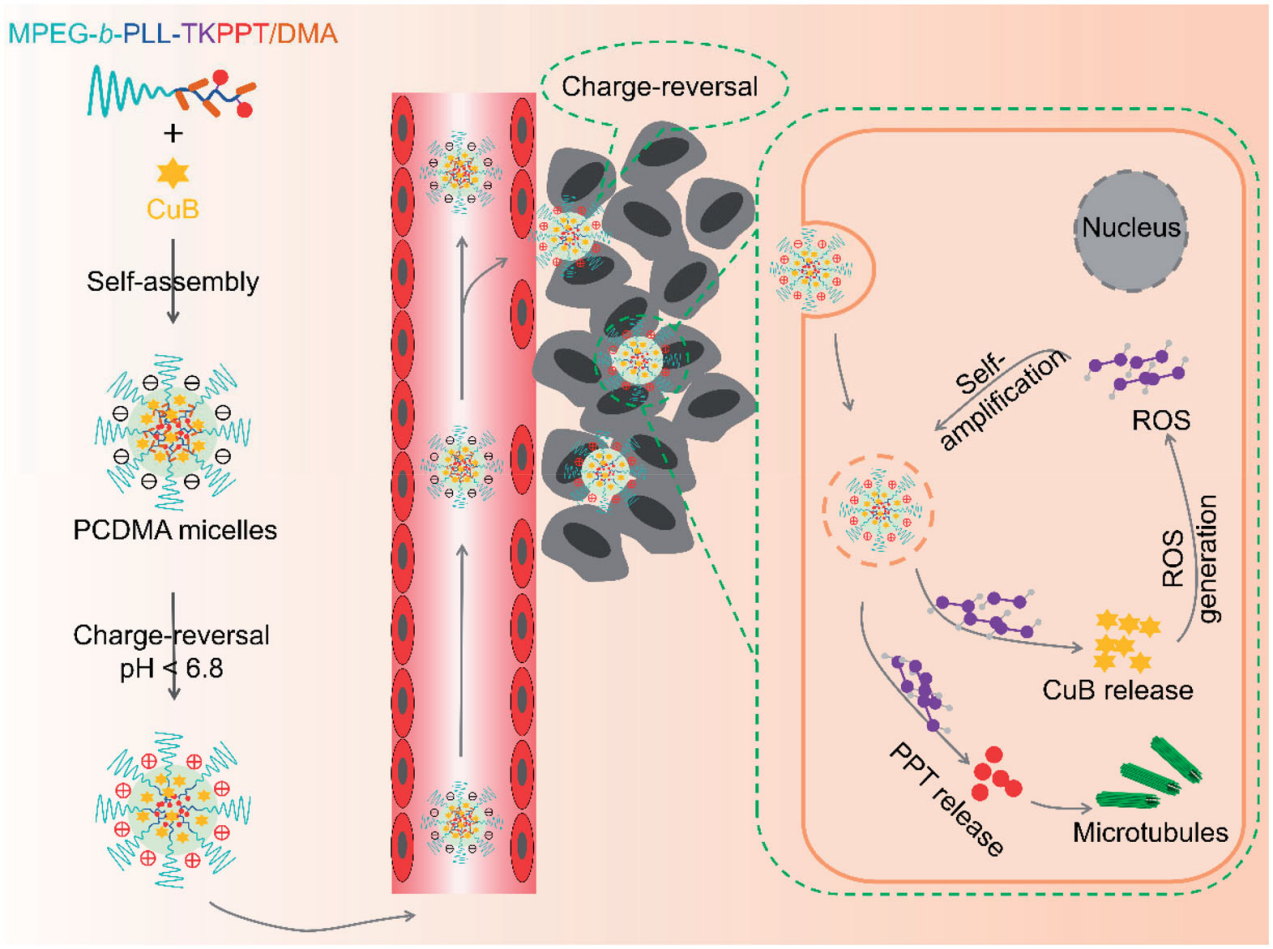
Illustration of the charge-conversion PCDMA system with self-amplifiable drug release for overcoming MDR *in vivo*.

Considering the above, in this study, a pH/ROS-responsive PPT-based PPM-DDS with charge-reversal and ROS generation was designed and prepared (PCDMA, Scheme 1) by encapsulating cucurbitacin B (CuB, a ROS generation agent) (Wang et al., 2020) into the pH/ROS-responsive PPT polymeric prodrug. PCDMA could maintian negative charge under blood circulation, which could transfer to positive charge after reach tumor tissue and promote the internalization of PCDMA by cancer cells. After enter cancer cells, endogenous ROS could trigger PPT and CuB release. The released PPT can effectively kill MDR cancer cells, and the CuB can produce abundant ROS, in turn to accelerate drug release, ultimately enhance treatment efficiency and overcome MDR.

## 2. Materials and methods

### 2.1. Synthesis of TK-PPT

TK-PPT was synthesized by an esterification reaction according to the previous report with modified (Xu et al., 2020). In brief, 414.4 mg PPT, 216.0 mg EDC, and 153.2 mg DMAP were dissolved in 25.0 mL of dry DMF and stirred at 40 °C for 16 h under a dry argon atmosphere. At the end of the reaction, the mixture was precipitated into excess ice-cold 0.01 M diluted hydrochloric acid and vacuum dried. The product was purified by high-performance liquid chromatography (HPLC) with a yield of 55.7%.

### 2.2. Synthesis of MPEG-*b*-PLL-TKPPT

Methoxyl poly(ethylene glycol)-*block*-poly((l-lysine)-*g*-(thioketal-podophyllotoxin)) (denoted as MPEG-*b*-PLL-TKPPT) was synthesized by conjugating TK-PPT to the amino group of MPEG-*b*-PLL. Typically, 970.5 mg TK-PPT, 172.5 mg NHS, and 270.0 mg EDC were dissolved in 40.0 mL dry DMF and stirred at room temperature for 2 h under a dry nitrogen atmosphere. Subsequently, the MPEG-*b*-PLL solution (900.0 mg in 20.0 mL of dry DMF) was added to the above mixture and stirred for a further 24 h at room temperature under an argon atmosphere. At end of the reaction, the reaction mixture was transferred into a dialysis bag (MWCO: 3500 Da) against DMF for 48 h to remove any unreacted small molecular impurities, and then against distilled water to remove any residual DMF. The MPEG-*b*-PLL-TKPPT was obtained after lyophilization (yield: 85.4%).

### 2.3. Synthesis of MPEG-*b*-PLL-TKPPT/DMA

Methoxyl poly(ethylene glycol)-*block*-poly((l-lysine)-*g*-(thioketal-podophyllotoxin)-*g*-(dimethylmaleic anhydride)) (denoted as MPEG-b-TKPPT/DMA) was synthesized by modifying DMA on the backbone of MPEG-b-TKPPT/DMA. Typically, 200.0 mg of MPEG-*b*-PLL-TK-PPT and 40.7 mg of DMA were dissolved in 10.0 mL of DMSO, then pyridine (0.1 mL) and triethylamine (0.1 mL) were added under an argon atmosphere. The mixture was stirred at room temperature for 24 h. After the reaction was completed, the mixture was dialyzed (MWCO: 3500 Da) against distilled water. The final product, MPEG-*b*-PLL-TKPPT/DMA was obtained by lyophilization (yield: 87.3%). As a control, succinic anhydride was grafted to MPEG-*b*-PLL-TKPPT using a similar procedure, and the obtained product was denoted as MPEG-*b*-PLL-TKPPT/SA (yield: 88.9%).

### 2.4. Preparation of micelles

Nanoprecipitation method was used to prepare the micelles. The micelles formed by MPEG-*b*-PLL-TK**P**PT/**DMA** and **C**uB were denoted as **PCDMA**; the micelles assembled by MPEG-*b*-PLL-TK**P**PT/**DMA** were abbreviated as **PDMA**, and the micelles formed by MPEG-*b*-PLL-TK**P**PT/**SA** and **C**uB were named as **PCSA**. Typically, 20.0 mg of PPT polymeric prodrug and 4.0 mg CuB were dissolved in 1.0 mL of DMSO and added into 10.0 mL of PBS dropwise under stirring. The DMSO and un-loaded CuB were removed through ultrafiltration, obtaining either PCDMA, PDMA, or PCSA. The concentration drug loading efficiency (DLE) and drug encapsulation efficiency (DEE) were measured by HPLC and calculated according to the following equation:

$$\mathrm{DLC}\;(\mathrm{wt}\%)\;=\;\frac{\text{weight}\;\text{of}\;\text{drug}\;\text{in}\;\text{micelles}}{\text{weight}\;\text{of}\;\text{micelles}}\;\times 100\%$$

$$\mathrm{DEE}\;(\mathrm{wt}\%)\;=\;\frac{\text{weight}\;\text{of}\;\text{drug}\;\text{in}\;\text{micelles}}{\text{weight}\;\text{of}\;\text{drug}\;\text{in}\;\text{feeding}}\;\times 100\%$$

Moreover, coumarin-6 loaded micelles were also prepared. Briefly, 5.0 mg MPEG-*b*-PLL-TKPPT/DMA (or MPEG-*b*-PLL-TKPPT/SA) and 50.0 μg coumarin-6 were dissolved in 1.0 mL of DMSO and added into 10.0 mL of PBS dropwise under stirring. Excess DMSO and coumarin-6 were removed through ultrafiltration, obtaining a coumarin-6 loaded micelle.

### 2.5. ROS-responsive assays

PCDMA micelles were incubated with PBS (pH 7.4) or PBS (pH 7.4) containing 10 mM H_2_O_2_ for 0, 3, 6, 9, or 12 h at 37 °C. At the end of incubation, the micelle size distribution was measured by dynamic lighting scattering (DLS).

### 2.6. *In vitro* drug release

A dialysis approach was employed to evaluate the drug release profiles of PCDMA. Briefly, 2.0 mL of freshly prepared PCDMA micelles were added to a dialysis bag and immersed into 48.0 mL of release medium. PBS (pH 7.4) with 0.1 H_2_O_2_, PBS (pH 7.4) with 10 mM H_2_O_2_, PBS at pH 7.4, PBS at 6.8, and acetic acid–sodium acetate buffer (pH 5.0) were used as the release medium. The release assay was performed in a thermotank at 37 °C with gentle shaking (100 rpm) under an argon atmosphere. After different incubation times, 1.0 mL of release solution was withdrawn and the amount of released PPT and CuB was measured by HPLC.

### 2.7. pH-induced zeta potential change of micelles

The PCDMA or PCSA micelle solution was diluted by PBS at pH 7.4 or 6.8 to the final concentration of 1.0 mg/mL and then incubated at 37 °C. After different incubation times, 1.0 mL of the micelle solution was withdrawn and the zeta potential of the solution was measured.

### 2.8. Cellular uptake

Cellular uptake of PCDMA and PCSA micelles at different pH conditions was measured using confocal laser scanning microscopy (CLSM, ZEISS LSM 780, Oberkochen, Germany) and flow cytometry (FCM, FACScan; Becton Dickinson, Franklin Lakes, NJ).

For CLSM analysis, the A549/PTX cell line was seeded in a glass-bottom dish at a density of 1.0 × 10^5^ cells per well and cultured overnight. Cells were treated with coumarin-6 loaded PCDMA or PCSA micelles at pH 7.4 or 6.8, respectively. After a 1-h or 4-h incubation, the cells were washed with RPMI 1640 twice, stained with DAPI for 10 min at room temperature, fixed with 4% formaldehyde for 10 min at room temperature, and then observed by CLSM.

For the FCM study, A549/PTX cells at a density of 1.0 × 10^5^ cells/well were seeded in six-well plates and cultured at 37 °C for 24 h. Next, cells were treated with the coumarin-6 loaded PCDMA and PCSA micelles for 1 h or 4 h in 2 mL complete RPMI 1640 at pH 7.4 or 6.8. At the end of the treatment period, cells were collected, washed, and assessed via FCM.

### 2.9. Intracellular ROS generation

ROS production in A549/PTX cells was detected by CLSM and FCM using dichlorofluorescein diacetate (DCFH-DA) as probe. For CLSM analysis, the A549/PTX cell line was seeded in a glass-bottom dish at a density of 1.0 × 10^5^ cells per well and cultured overnight. Cells were treated with CuB, PDMA, PCDMA, or PCSA micelles for 4 h and then incubated with FBS free RPMI 1640 containing DCFH-DA for 30 min. Next, cells were washed with RPMI 1640 and then observed by CLSM. For FCM analysis, cells were seeded onto six-well plates and grew for 24 h. After incubation with CuB at different concentration (0.01, 0.1, 1, 2, 5, or 10 μg/mL) for 2 h or of 0.1 μg/mL CuB at different incubation times (2, 4, 6, 8, 10, or 12 h), cells were incubated with DCFH-DA for 30 min. Subsequently, cells were digested by trypsin, collected through centrifugation, washed with RPMI 1640, and then conducted on an FCM.

### 2.10. Intracellular self-amplification PPT release

To monitor intracellular self-accelerated drug release, A549/PTX cells seeded on six-well plates were treated with PCDMA or PCSA micelles for 8 h, 12 h, or 24 h, respectively. At the end of treatment, cells were washed with cold PBS, trypsinized, and collected into a tube. The tube was placed in a thermostatic ice bath at 0 °C and then sonicated the cell suspension with alternative cycles of 5 s pluses after every 10 s intervals for 5 min using ultrasonicator probe (SCIENT-IID). Subsequently, the mixture was mixed with chloroform/methanol (4:1, v/v) to extract the released PPT or PPT prodrug. The concentration of PPT was measured by HPLC as mentioned above.

### 2.11. Cytotoxicity evaluation

The cytotoxicity of drugs was evaluated by MTT assays. Typically, A549/PTX and A549 cells were seeded into 96-well plates at a density of 5000 cells/well. After culturing overnight, cells were treated with paclitaxel (PTX), PPT, or PCDMA at pH 7.4 for 48 h. At the end of the incubation period, 20 μL of an MTT solution (5 mg/mL) was added to each plate. After another 4 h, the medium was removed and 200 μL of DMSO was added to dissolve the crystals, and the absorbance was detected at a wavelength of 490 nm using a Bio-Rad 680 microplate reader (Hercules, CA). Cell viability was calculated as follows: cell viability (%)=*A*_sample_/*A*_control_×100%, where ‘*A*_sample_’ and ‘*A*_control_’ represented the absorbance of sample and control wells, respectively.

To determine the influence of pH on cytotoxicity of micelles, A549/PTX cells were incubated with PCDMA or PCSA for 4 h at pH 7.4 or 6.8. After treatment, the culture medium was replaced with fresh RPMI 1640, and then subjected to the MTT assay after incubation for 44 h.

To determine the influence of ROS on cytotoxicity of micelles, A549/PTX cells were incubated with PCDMA or PDMA for 48 h at pH 7.4, and then subjected to the MTT assay.

The IC_50_ value of all drug formulation was calculated using the software of GraphPad Prism 6 (GraphPad Software, La Jolla, CA). The resistance index (RI) was calculated as: RI=(IC_50_ of resistant cell)/(IC_50_ of sensitive cell) according to previous report (Roy et al., 2015).

### 2.12. Maximum tolerated dose evaluation

Kunming mice were used as the animal model to study the maximum tolerated dose (MTD) of free PPT and its polymeric prodrug micelles according to previously published protocols (Zhou et al., 2018). Typically, mice were divided into 17 groups at random with 10 mice in each group and treated with PCDMA micelles (equivalent to PPT at 20, 50, 100, 150, 200, 250, or 300 mg/kg), PDMA micelles (equivalent to PPT at 20, 50, 100, 150, 200, 250, or 300 mg/kg), PCSA micelles (equivalent to PPT at 20, 50, 100, 150, 200, 250, or 300 mg/kg), or free PPT (5, 10, 15, 20, and 30 mg/kg), respectively, administered via the tail vein. The survival and body weight of mice were recorded daily for 14 days and stop recording when a mouse died. The drug at its maximum dose that did not result in animal death or no more than a 20% body weight decrease within the entire period, was identified as the MTD.

### 2.13. *In vivo* antitumor study

The *in vivo* antitumor effect of the preparations was evaluated in an A549/PTX tumor-bearing nude mouse model. The A549/PTX tumor-bearing nude mouse model was established by injecting 5.0 × 10^6^ cells in 100.0 μL PBS into the right flanks of BALB/c nude mice. Mice were randomly divided into four groups when the tumor volume reached about 80–100 mm^3^ and each group contained six mice. The mice have administrated drugs via the tail vein in a single injection (15.0 mg/kg for free PPT; 15.0 mg/kg for PCDMA, PDMA, and PCSA; and 200.0 mg/kg for PCDMA, PDMA, and PCSA), and this day was recorded as day 0. The body weight and tumor size were recorded every two days for a total of 14 days. Tumor volume (*V*) was measured by recording width (*W*) and length (*L*), and calculated as follows:

$$V\;=\;\frac{\mathrm{LW}2}{2}$$

On day 14, the mice were euthanized and the tumors were collected. The tumor tissues were weighed to measure the tumor suppression ratio (TSR), which is calculated according to the following equation:

$$\mathrm{TSR}\;(\%)\;=\;\frac{W_{\mathrm{sample}}-W_{\mathrm{saline}}}{W_{\mathrm{saline}}}\;\times \;100\%$$

where *W*_sample_ and *W*_saline_ indicated the tumor mass of the sample groups and saline group.

### 2.14. Statistical analysis

Data are reported as mean ± standard deviation (SD). Two-tailed Student’s *t*-test or one-way ANOVA was employed for statistical analysis using the SPSS version 19 statistical software (SPSS, Chicago, IL). Statistical significance was denoted as *p< .05.

## 3. Results and discussion

### 3.1. PPT prodrug designed and self-assembly

The synthetic protocol for MPEG-*b*-PLL-TKPPT/DMA is illustrated in Fig. S1. First, MPEG-*b*-PLL and TK-PPT were synthesized; then, TK-PPT was grafted onto the amino group of MPEG-*b*-PLL to obtain MPEG-*b*-PLL-TKPPT; finally, DMA was conjugated to MPEG-*b*-PLL-TKPPT to obtain MPEG-*b*-PLL-TKPPT/DMA. MPEG-b-PLL was selected as the micelle material because of its low toxicity, excellent biocompatibility, and complete biodegradability (Lv et al., 2014; Luan et al., 2019; Xu et al., 2020). The polyamine acid chain can be decomposed by enzymatic hydrolysis of the peptide bond, and the degradation product can be applied to the life cycle of the organism (Guo et al., 2020).

The TK linker and TK-PPT were synthesized first and confirmed by ^1^H NMR and mass spectrometry (MS), in detail (Figs. S2–S3). Both the ^1^H NMR spectrum and MS spectrum confirm that TK and TK-PPT were successfully synthesized. Subsequently, MPEG-NH_2_ induced the Lys(Z)-NCA ring-opening polymerization and acid-mediated deprotection of the benzyl group to obtain MPEG-*b*-PLL. ^1^H NMR was employed to measure the structure of MPEG-*b*-PLL (Fig. S4). The peaks in the spectra were well assigned and the results were consistent with previous reports (Wang et al., 2016), demonstrating the successfully preparing MPEG-*b*-PLL. After comparing the peak area of the methylene protons of PEG with Lys methylene protons, the degree of polymerization (DP) of MPEG-*b*-PLLZ and MPEG-*b*-PLL was calculated, which was 30 for both polymers. To further measure the DP of PLL, gel permeation chromatography (GPC) was used to determine the molecular weight of the polymers. As presented in Table S1, the GPC results also revelated that the DP of Lys was 30 in these two polymers. The TK-PPT was subsequently conjugated to MPEG-*b*-PLL to obtain the PPT polymeric prodrug MPEG-*b*-PLL-TKPPT, which was analyzed by ^1^H NMR spectrum, UV spectra, and GPC in detail (Fig. S5 and S6, and Table S1), and these results verified that MPEG-*b*-PLL-TKPPT was successfully synthesized. Finally, DMA was reacted with free amino groups in MPEG-*b*-PLL-TKPPT to acquire the charge-reversion polymer: MPEG-*b*-PLL-TKPPT/DMA. The ^1^H NMR spectrum (Fig. S5) and GPC assay (Table S1) showed that DMA was successfully conjugated. The control prodrug, MPEG-*b*-PLL-TKPPT/SA, with no charge-transfer capability was synthesized and confirmed by GPC (Table S1).

**Figure 1.**
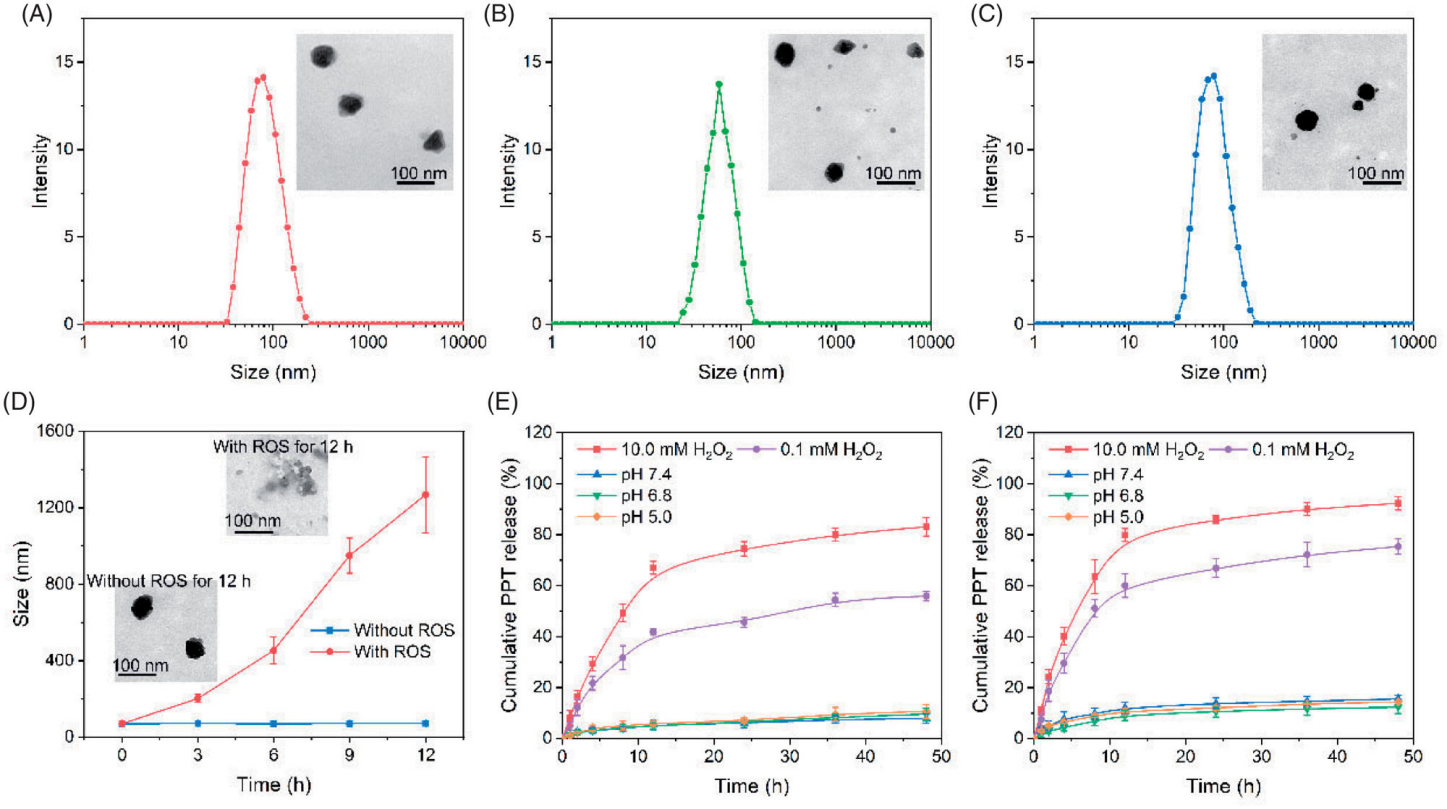
Characterization of micelles. The size distribution of PCDMA (A), PDMA (B), and PCSA(C) in PBS at pH 7.4. (D) Size changes of PCDMA after incubation with or without 10 mM H_2_O_2_ for different time. PPT (E) and CuB (F) release from PCDMA under different ROS conditions or pH conditions. Data are shown as mean ± SD, *n*= 3.

The two amphiphilic block copolymer prodrugs, MPEG-*b*-PLL-TKPPT/DMA and MPEG-*b*-PLL-TKPPT/SA, can self-assemble into polymeric micelles in an aqueous solution. Critical micelle concentration (CMC) value of MPEG-*b*-PLL-TKPPT/DMA and MPEG-*b*-PLL-TKPPT/SA detected using Nile Red as the probe was as low as 18.5 and 16.7 μg/mL, respectively (Fig. S7). Subsequently, the hydrophobic drug CuB was encapsulated into the PPT prodrug via the nanoprecipitation method. To well investigate our hypothesis, three micelles: PCDMA (charge-reversal, ROS-response, and ROS-generation), PDMA (ROS-response and charge-reversal), and PCSA (ROS-response and ROS-generation) were prepared in detail (Table S2). Dynamic light scattering (DLS) and transmission electron microscope (TEM) were applied to characterize the physicochemical properties of the three micelles (Figure 1(A–C) and Table S3). The hydrodynamic size of PCDMA, PDMA, and PCSA micelles was (70.1 ± 2.9), (63.8 ± 2.3), and (70.9 ± 3.9) nm, respectively, all smaller than 100 nm and with a narrow distribution. The ideal characteristic sizes would enable the micelles to be easily accumulated in the tumor tissues by the EPR effect (Wang et al., 2016). The TEM micrograph demonstrated that the micelles were near spherical in shape. The DLE of PPT in PCDMA, PDMA, and PCSA measured by HPLC was (19.4 ± 1.2)%, (23.4 ± 1.3)%, and (20.4 ± 1.4)%, respectively, and the DLE of CuB in PCDMA and PCSA was (4.7 ± 0.6)% and (5.0 ± 0.5)%. Additionally, the three micelles have good stability in PBS and RPMI 1640 with 10% FBS (Fig. S8) over 24 h. The low CMC value and good structure stability may be beneficial to overcome the dilution effect in blood circulation and improve drug delivery (Dai et al., 2019).

### 3.2. ROS-responsive capability evaluation

Size changes and *in vitro* drug release were investigated to evaluate the ROS-responsive ability of PCDMA micelle. As shown in Figure 1(D), the size of PCDMA micelles had no remarkably changed in the absence of ROS. On the contrary, while the PCDMA micelles were incubated with 10.0 mM H_2_O_2_, its size rapidly disintegrated and changed from 70 nm to 1280 nm within 12 h. The TEM results were well consisted with DLS results. The underlying mechanism is that the TK-linker between PPT and polymer backbone could cleave in response to ROS (Yin et al., 2019), and subsequently induce the hydrophobic core of PCDMA transfer to a hydrophilic surface, leading to the disassembly of micelles. To further confirm this mechanism, the drug release behavior of PCDMA in various ROS conditions was analyzed. As shown in Figure 1(E), in the absence of H_2_O_2_ (pH 7.4), only a small quantity of PPT (below 8%) was released from PCDMA within 48 h, demonstrating the good stability of micelles in normal conditions, which is beneficial to avoid the side effects due to the inadequate PPT release in normal tissues. When the H_2_O_2_ increased to 0.1 mM, approximately 55.9% PPT was released from the PDMA micelles. Notably, the release percentage of PPT reached 83.1% upon exposure to 10.0 mM H_2_O_2_. Moreover, CuB is released rapidly even at a low ROS level (Figure 1(F)), which may be due to the non-covalent interactions between polymers and CuB. Furthermore, CuB release is drastically enhanced with the increase of ROS dose. Reasonably, a high level of ROS can remarkably accelerate PPT release, resulting in the micellar structure changed to loosen and unstable, ultimately leading to enhanced CuB release. These results further confirmed the ultra-sensitive of PCDMA to ROS. Additionally, the drug release at different pH conditions was also evaluated. It was observed the total released of CuB had no significant difference between various pH values within 48 h (Figure 1(F)). Moreover, even the pH value fell to below 5.5, the cumulative release of PPT did not exceed 11% within 48 h, suggesting that the PPT prodrug micelles could maintain stability under physiological and pathological pH conditions (Figure 1(E)).

### 3.3. pH-activated charge-transition enhances cell uptake

**Figure 2.**
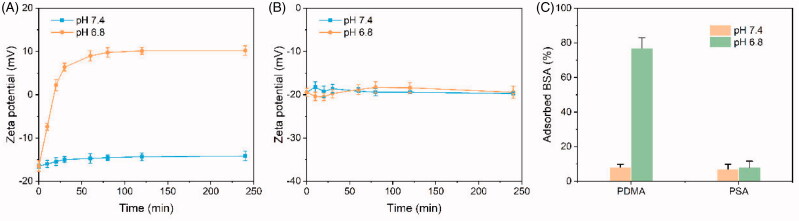
Charge-switchable PCDMA micelles. Surface zeta potential changes of PCDMA (A) and PCSA (B) at pH 7.4 or 6.8 at different incubation times. (C) The amount of BSA adsorbed on the PCDMA and PCSA micelles after incubation at pH 7.4 or 6.8 (*n*= 6, ***p< .001).

One of the major properties of PCDMA micelles is the tumor tissue weakly acidic-mediated charge-conversion. The potential changes of PCDMA to the surface charge at pH 7.4 and 6.8 were determined to evaluate the pH-dependent charge-switching properties. As illustrated in Figure 2(A), the zeta potential of PCDMA under physiological conditions (pH 7.4) was maintained by a strong negative charge and only negligibly changed within a 4-h incubation period. When the pH decreased to pH 6.8 (the tumor tissue condition), the zeta potential of the PCDMA micelle significantly shifted from negative (–16.5 mV) to positive (10.2 mV) after 4-h incubation. In comparison, the control PCSA micelle remained strongly negatively charged with slight changes when incubated at pH 7.4 or 6.8 for 4 h (Figure 2(B)). The underlying reason can be explained as follows: in the weak condition, the distal carboxyl group of the DMA in the proximity of an amide bond and the rigid conformation due to neighboring double bonds resulted in intramolecular catalysis, in turn, promoting rapid DMA hydrolysis (Fig. S9) (Feng et al., 2016). However, the SA product presents no double bonds, thus led to a flexible conformation that remained stable under weakly acidic conditions (Feng et al., 2016). To further investigate the pH-responsive charge-switchable process of PCDMA, the adsorption of BSA at different pH conditions was measured. As illustrated in Figure 2(C), in the PCSA group, only a small number of BSA molecules was adsorbed both at pH 7.4 and 6.8 within 12 h. Conversely, over 70% of BSA was adsorbed onto the PCDMA at pH 6.8 within 12 h, while only 8.1% of the BSA adsorption was determined at pH 7.4 over the same duration. These results indicated that the PCDMA could avoid protein adsorption during blood circulation.

**Figure 3.**
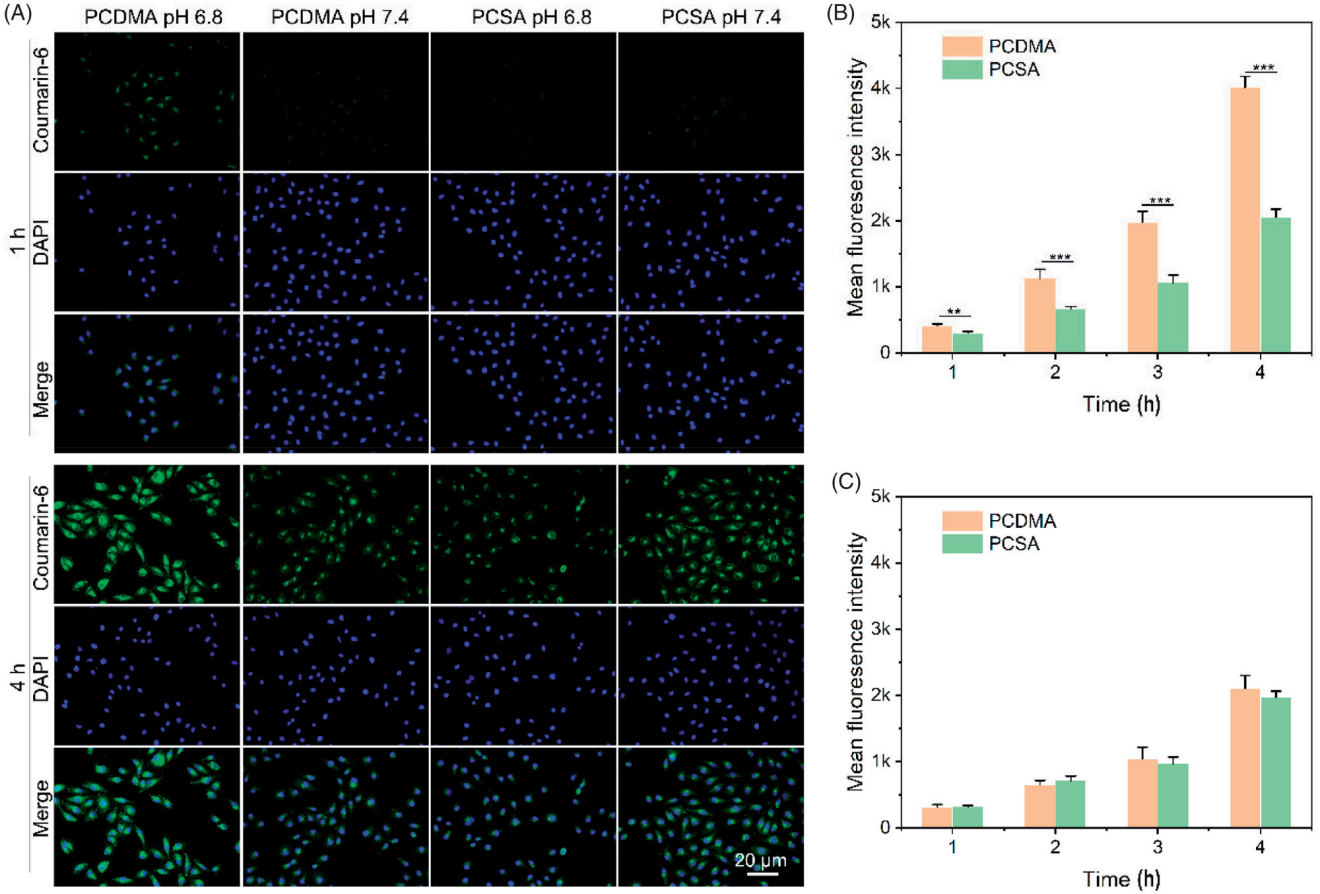
(A) CLSM images of A549/PTX cells after treatment with PCDMA and PCSA at pH 7.4 or 6.8. FCM results of A549/PTX cells after treatment with PCDMA and PCSA at pH 6.8 (B) or 7.4 (C). *n*= 6, ***p< .001.

It is well known that the micelles with a positive charge could promote their attachment to the negatively charged cell membrane (Feng et al., 2020). The above studies demonstrated that the surface charge of PCDMA may rapidly be switched from negative to positive under weakly acidic conditions, which may promote PCDMA internalization by cancer cells. The cell uptake assay was performed to confirm this phenomenon. PCDMA micelles internalized by A549/PTX cells were detected by CLSM and FCM using coumarin-6 as the fluorescence probe. As shown in Figure 3(A), in the CLSM images, the cellular uptake of micelles was time-dependent mode under pH 7.4 or 6.8. In the PCSA treatment group, the green fluorescence signal of coumarin-6 in cells was not different between pH 7.4 and 6.8 within the same duration. Similarly, the fluorescence intensity of the PCDMA treated group and PCSA group at pH 7.4 was also not significantly different. However, after incubation at pH 6.8, the intracellular green fluorescence signal in the PCDMA-treated group was significantly stronger than that of PCDMA at pH 7.4 and PCSA 6.8. These results indicated that more PCDMA micelles were internalized by cancer cells in weakly acidic conditions. The FCM results were well consistent with the CLSM results (Figure 3(B,C)). At pH 6.8, the mean fluorescence intensity (MFI) in the PCDMA group was 1.4-, 1.7-, 1.9-, and 2.0-fold higher than that of the PCSA group after incubation for 1, 2, 3, and 4 h, respectively (Figure 3(B)). There was no difference between PCDMA and PCSA at pH 7.4 (Figure 3(C)). These results demonstrated that the PCDMA micelles under weakly acidic conditions, induced by their surface charge switch, could promote their internalization by cancer cells.

### 3.4. Intracellular ROS generation and amplification drug release

**Figure 4.**
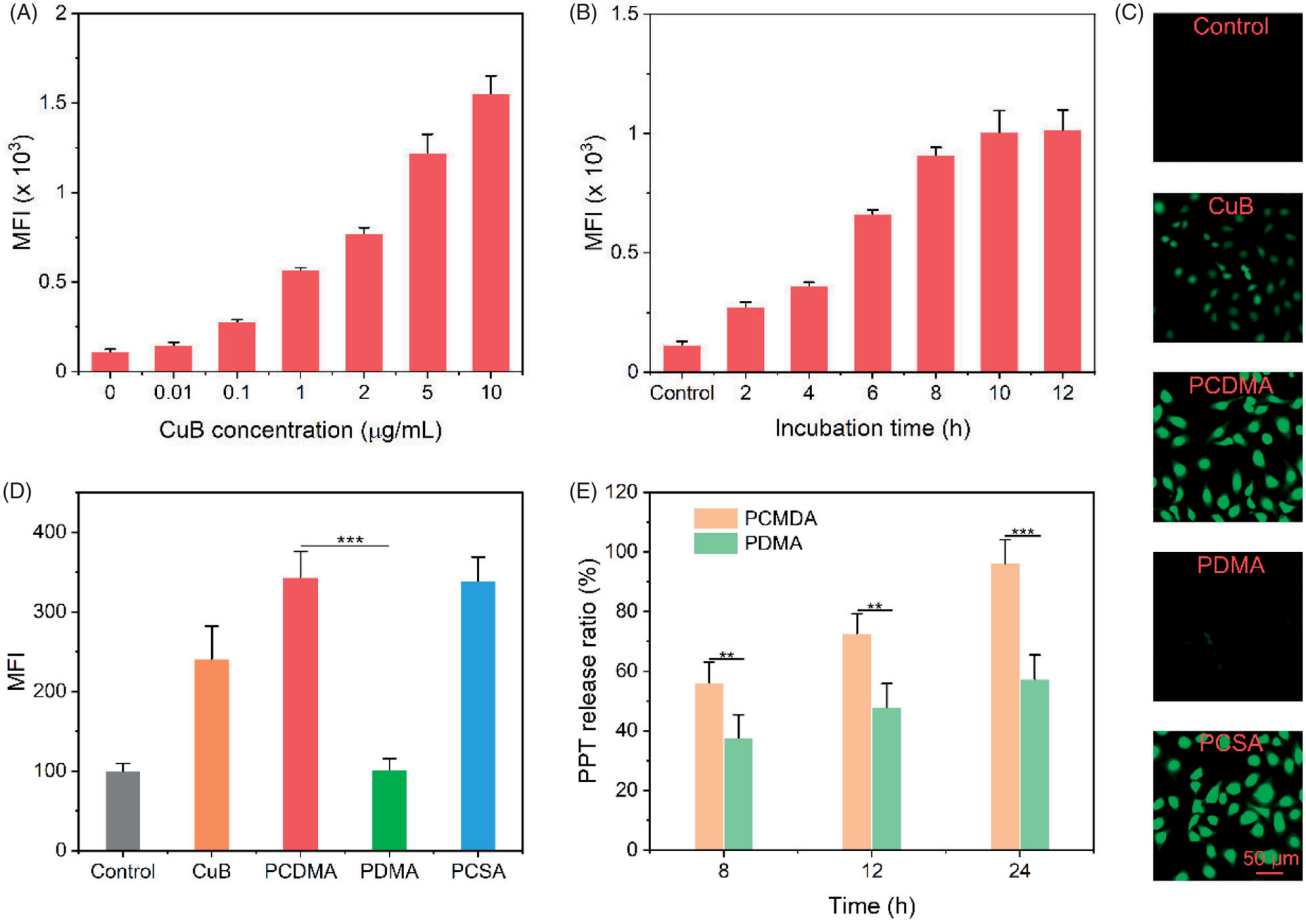
Evaluation of the ROS regenerating ability of CuB *in vitro* and intracellular self-amplification drug release of PCDMA. FCM results of A549/PTX cells treated with CuB for different concentration for 2 h (A) or 0.1 μg/mL of CuB for different incubation time (B), *n*= 6. CLSM images (C) and FCM analysis (D) of A549/PTX cells stained with DCFH-DA after treatment with blank culture medium, CuB, PCDMA, PDMA, or PCSA for 4 h (*n*= 6, ***p< .001). (E) PPT release amount of PCDMA and PDMA in A549/PTX cells after 8, 12, and 24 h incubation (*n*= 3, **p< .01; ***p< .001).

After internalized by cancer cells, PPT should completely release from PCDMA to provide sufficient PPT to kill MDR cancer cells. However, efficient drug release would be very difficult for PCDMA under the trigger of intrinsic ROS level in tumor tissues. CuB, a typical tetracyclic triterpenoid compounds, is isolated from *Trichosanthes kirilowii Maximowicz* (Sun et al., 2015). The emerging evidence shows that CuB has the great potential to inhibit lung cancer, breast cancer, cervical carcinoma, and colon cancer by remarkably increasing intracellular ROS levels (Yasuda et al., 2010; Zhang et al., 2012; Guo et al., 2014; Ren et al., 2015). Wang et al. demonstrated that CuB can generate massive ROS in MDA-MB-231 cells to amplify the degradation of a ROS-sensitive PTX prodrug (Zhou et al., 2018). Encouraging by the excellent intracellular ROS production ability of CuB, it was selected as a ROS generator to promote intracellular PPT release in this study. To test our hypothesis, the intracellular ROS augmentation capability of CuB against A549/PTX cells was monitored using the ROS probe 2′,7-dichlofofluoresceindiacetate (DCFH-DA) at first. Flow cytometry analysis was performed to quantitatively investigate the ROS production ability of CuB. As exhibited in Figure 4(A,B), the MFI in cells was enhanced with the increased CuB concentration (Figure 4(A)) or the prolonged incubation time (Figure 4(B)), which demonstrated the intracellular ROS generation capability of CuB. Subsequently, the ROS producing ability of CuB loaded PPM-DDS within A549/PTX cells was further evaluated by CLSM and FCM. As shown in Figure 4(C), the green fluorescence signal of dichlorofluorescein (DCF, the oxidative product of DCFH-DA) in the CuB, PCDMA, and PCSA treatment group was significantly higher than that of PBS and PDMA incubation group. Moreover, FCM analysis illustrated that the cells treated with CuB, PCDMA, and PCSA enhanced the MFI about 1.3-/1.4-, 1.4-/1.6-, and 1.7-/1.8-times in compared with PBS and PDMA group, respectively (Figure 4(D)). The qualitative analysis and quantitative analysis both proved that the intracellular ROS generation capability of CuB-loaded PPM-DDSs.

To validate whether the increased ROS in cancer cells could improve the PPT release, the released PPT in cancer cells after treated with PCDMA and PDMA was extracted and detected by HPLC. As presented in Figure 4(E), very low free PPT in the PDMA group can be observed, and only 64% (per 10^6^ cells) of PPT was released from cell internalized PDMA even incubation for 24 h. On the contrary, in the PCDMA group, free PPT was sharply higher than the PDMA group, where the release of PPT from cell internalized PCDMA was 1.5-, 1.5-, and 1.7-fold over than PDMA after treatment for 8, 12, and 24 h, respectively. In a word, CuB loaded PPT-based PPM-DDS was confirmed to increase intracellular ROS level efficiently in A549/PTX cells, which can further promote ROS-responsive drug release efficiency.

### 3.5. Evidence of overcoming of MDR *in vitro*

**Figure 5.**
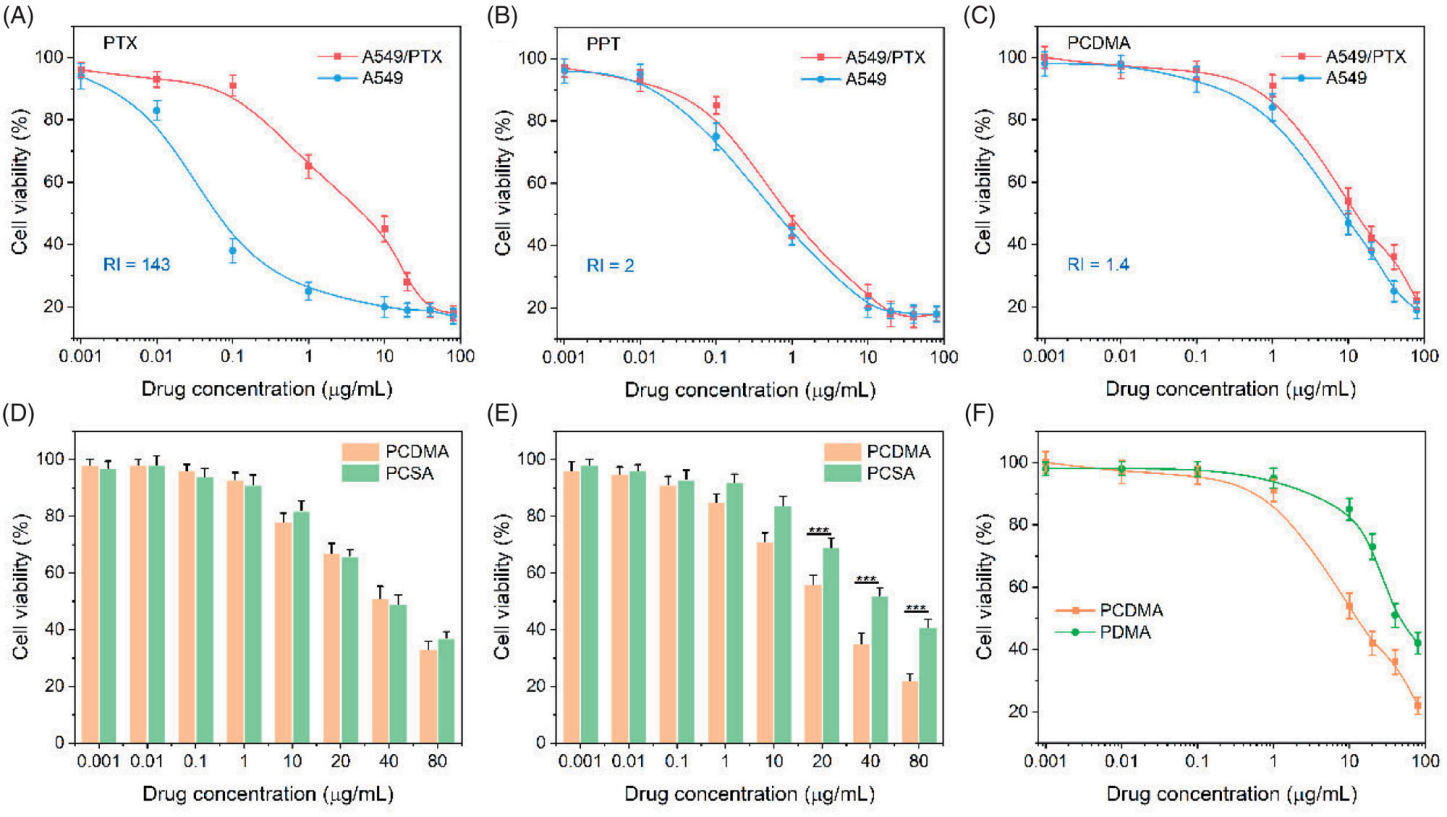
*In vitro* cytotoxicity assay. Cell viability of A549/PTX and A549cells after treatment with PTX (A), PPT (B), or PCDMA (C) at pH 7.4 for 48 h. Cell viability of A549/PTX following treatment with PCDMA and PCSA at pH 7.4 (D) and 6.8 (E) for 4 h. (F) Cell viability of A549/PTX following treatment with PCDMA and PDMA at pH 7.4 for 48 h. *n*= 6, ***p< .001.

Overcoming of the MDR effect *in vitro* by PPT was investigated using MTT assays in MDR cancer cell lines (A549/PTX cells) and corresponding normal cancer cells (A549 cells). As shown in Figure 5(A,B), and Table S4, the RI of PTX was 143, confirming the A549/PTX cells were sharply resistant to PTX. In contrast, the RI of PPT against A549/PTX and A549 cells was 2, suggesting that PPT can effectively inhibit the MDR cancer cell lines *in vitro*. This result was well consistent with previous reports (Roy et al., 2015; Zhou et al., 2018). Similarly, PCDMA could also effectively suppress the growth of the two cell lines with a small RI (1.4) (Figure 5(C) and Table S4), indicating that PCDMA can also combat MDR.

Moreover, the results of the cellular uptake study described above demonstrated that PDMA could quickly be internalized by cancer cells under weakly acidic conditions, which may increase their anticancer effects. This phenomenon was investigated by the MTT assay at pH 7.4 and 6.8 using A549/PTX cells. Cells were treated with PCDMA and PCSA at pH 7.4 or 6.8 for 4 h and then cultured in a drug-free medium for an additional 44 h. The cell growth inhibition effect of PCDMA and PCSA was not different at pH 7.4 (Figure 5(D)). The cell viabilities of A549/PTX cells treated with PCDMA and PCSA at pH 7.4 were all above 65% at all the same drug concentration. In contrast, when the pH value was reduced to 6.8, the cell viabilities in the PCDMA treatment group were significantly lower than the PCSA-treated group when the PPT concentration was over 10.0 μg/mL (Figure 5(E)). Furthermore, the cell viabilities treated with PCSA at pH 7.4 and 6.8 at all drug concentrations were higher than 65%. However, PCDMA in the pH 6.8 condition exhibited a higher proliferation inhibition than that observed pH 7.4. These results indicated that the pH-sensitive charge-switchable PCDMA micelles more effectively inhibited the proliferation of cancer cells at pH 6.8 than that at pH 7.4, and it also indicated a more effective inhibition of proliferation of cancer cells at pH 6.8 than the non-charge-reversal PCSA micelles.

Moreover, cytotoxicity of PCDMA and PDMA against A549/PTX cells was also examined using MTT method. As presented in Figure 5(F), it can be easily observed that the PDMA did not exhibit noticeable cytotoxicity against A549/PTX cells, which may be due to the insufficient drug release under the initial relatively low ROS concentration in A549/PTX cells. As expected, the PCMDA showed conspicuous cytotoxicity against A549/PTX cells due to the self-accelerating drug release mode and the synergistic effects of PPT and CuB.

### 3.6. Maximum tolerated dose

**Figure 6.**
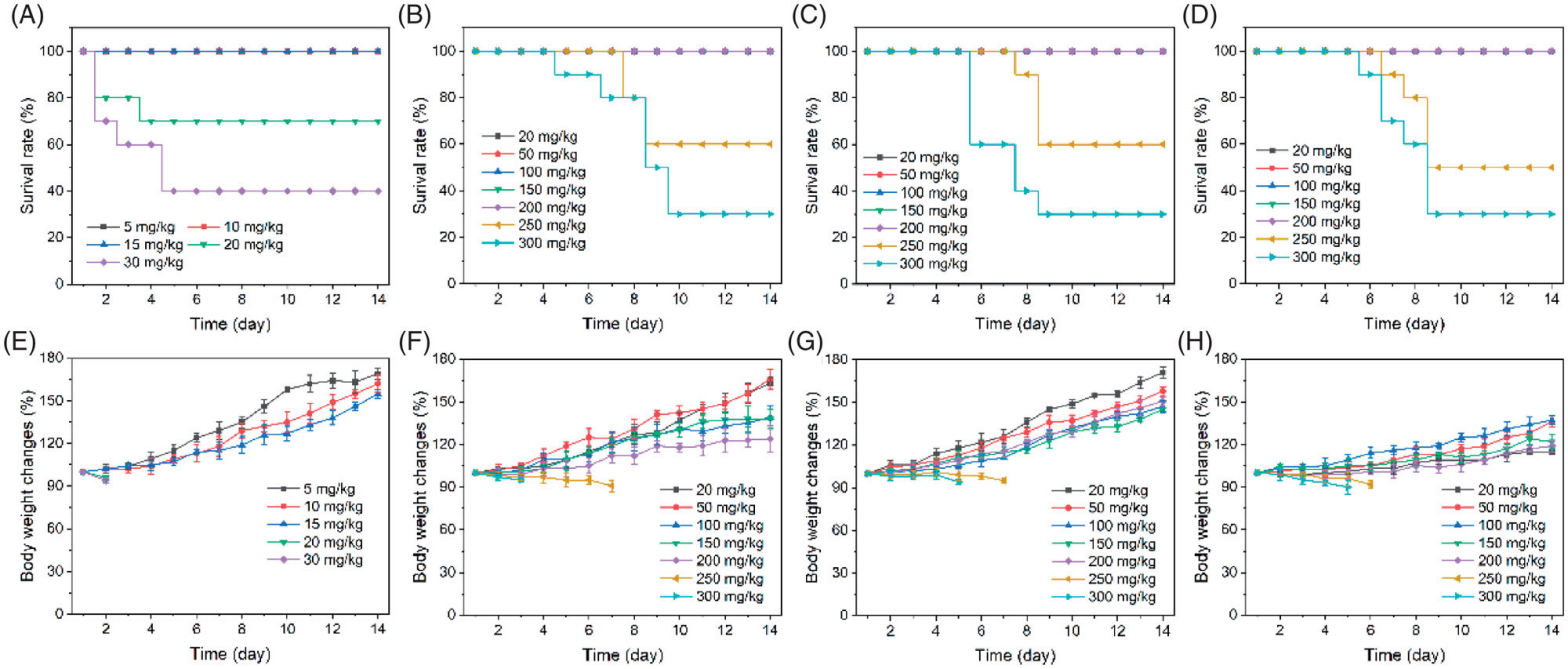
MTD study. The survival rate of Kunming mice after treated with free PPT (A), PCDMA (B), PDMA (C), and PCSA (D). The body changes of Kunming mice after treated with free PPT (E), PCDMA (F), PDMA (G), and PCSA (H). *n*= 10.

It has been reported that free PPT could immediately produce toxicity to the body after administration and could lead to death in mice within one or two days (Zhou et al., 2018). Previous study demonstrated that the free PPT has a low MTD (about 15 mg/kg) in Kunming mice (Li et al., 2019). In contrast, the polymeric PPT-prodrug usually had a higher MTD, because of the slow release kinetics of PPT under physiological conditions (Feng et al., 2020). As mentioned above, the release of PPT from PCDMA micelles in the absence of ROS was lower than 15%, thus, PCDMA may increase the MTD of PPT. Kunming mice without tumors were used as the animal model to assess the MTD of all PPT formulations. Mice were administered PPT-prodrug micelles or free PPT intravenously at different doses, and then, the toxicity-induced death and body weight of mice were recorded daily. As illustrated in Figure 6, no mice deaths or serious body weight losses were observed when the dose of free PPT was 15 mg/kg, and two mice died when the free PPT dose reached 20 mg/kg, indicating that the MTD of free PPT was 15 mg/kg. In contrast, the MTD of PCDMA, PDMA, and PSA micelles reached up to 200 mg/kg (equal to that of PPT), which is 13.5-fold higher than free PPT. The proposed underlying mechanism is that the TK linkage is stable in the physical environment, thereby, all PPT-prodrug micelles possessed sustained or non-PPT release properties, and produced a weak side effect. The high MTD allows PPT-prodrug micelles to be used at higher doses and will allow enhancing its antitumor efficiency.

### 3.7. *In vivo* antitumor efficacy

**Figure 7.**
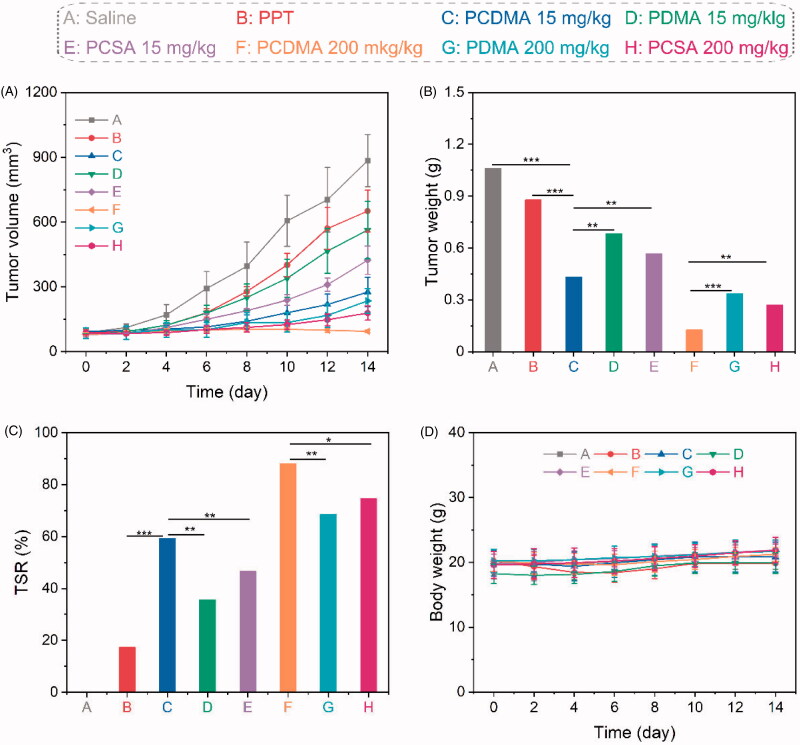
*In vivo* antitumor efficiency. Tumor volume changes (A), tumor weight at day 14 (B), TSR (C), and body weight changes (D) during the study. *n*= 6, **p< .01, ***p< .001.

Encouraged by the excellent capability of PCDMA to inhibit the proliferation of MDR cancer cells, we further investigated its antitumor activity *in vivo*. A549/PTX tumor-bearing mice received a single treatment with saline, free PPT (15 mg/kg), PCSA (15 mg/kg, equal to PPT), PDMA (15 mg/kg, equal to PPT), PCDMA (15 mg/kg, equal to PPT), PCSA (200 mg/kg, equal to PPT), PDMA (200 mg/kg, equal to PPT), or PCDMA (200 mg/kg, equal to PPT). Tumor volumes and body weight were recorded every two days and the tumor weight was measured at the end of the experiment. As shown in Figure 7(A–C), the tumor size in the PBS group grows quickly and the tumor volumes increased to 10.2-fold higher than day 0 after 14 days suggesting A549/PTX cells are highly aggressive. Additionally, with the treatment of free PPT, the tumor volume still growth to 7.6-fold, and the tumor weight-based TSR only 17.3%, indicating poor tumor suppression. Poor solubility and low bioavailability of small molecules may be the reason for limited therapeutic efficacy. Notably, PCDMA, PDMA, and PCSA exhibited significantly better antitumor activity compared with free PPT at the same dose with 2.9-, 7.1-, and 5.4-fold tumor volume increase, respectively. The TSR of PCDMA, PDMA, and PCSA was 59.4%, 35.7%, and 46.6%, respectively. Interestingly, PCDMA, PDMA, and PCSA micelles showed persistent tumor growth inhibitory effect with the TSR was higher at 88.1%, 68.5%, and 74.6%, respectively, when used at their MTD dose. Moreover, significantly more antitumor activity was found in the PCDMA micelles compared to the same dose in both the PDMA and PCSA group, demonstrating the combination of charge-reversal and intracellular self-amplification drug release strategy could significantly improve drug delivery efficacy. It should be noted that no remarkable body weight loss or adverse effects were observed for all groups during the treatment (Figure 7(D)), suggesting the toxicity of all PPT formulations has negligible toxicity. The superior biosafety of PCMDA would be a great advantage for their clinical translation. Taken together, charge-reversal-mediated high cellular uptake, intracellular efficient ROS production, and ROS-triggered rapid and complete drug release lead to excellent antitumor efficacy and low toxicity of PCMDA micelles.

## 4. Conclusions

In summary, we developed a novel well-defined pH and ROS subsequent-responsive PPM-DDS with surface charge-reversal and self-amplifiable drug release for MDR lung cancer treatment. The comprehensive *in vitro* and *in vivo* studies demonstrated that PCDMA not only effectively enhanced cell uptake via tumor acidity-triggering charge switching and ROS-activating drug release but also efficiently replenished the intracellular ROS to achieve rapid and complete drug release to overcome MDR. Moreover, it dramatically improves the MTD dose of free PPT, resulting in significantly enhancing tumor treatment efficacy with low toxicity *in vivo*. The work provides a promising nanomedicine with superior antitumor activity.

## Supplementary Material

Supplemental Material
