# Supplementary material for: pH and ROS sequentially responsive podophyllotoxin prodrug micelles with surface charge-switchable and self-amplification drug release for combating multidrug resistance cancer
Source: Drug Deliv. 2021 Apr 5;28(1):680–91. doi: 10.1080/10717544.2021.1905750 (PMC8023596; doi:10.1080/10717544.2021.1905750)
Supplement: Supplemental Material [file IDRD_A_1905750_SM2344.docx]

Supporting Information

**pH and ROS sequentially-responsive podophyllotoxin prodrug micelles with surface charge-switchable and self-amplification drug release for combating multidrug resistance cancer**

Chao Li^1,#^, Yifan Wang^2,#^, Shuo Zhang^1^, Jiaojiao Zhang^1^, Fang Wang^1^, Yunhao Sun^3^, Lirong Huang^3,*^, Wen Bian^3,*^

1. Department of Infectious Disease, Wuhu NO.1 People’s Hospital, Wuhu, 241060, China.

2. Department of Oncology, Yancheng NO.1 People’s Hospital, Yancheng First Hospital Affiliated Hospital of Nanjing University Medical School, Yancheng, 224005, China.

3. Department of Cardiothoracic Surgery, Yancheng NO.1 People’s Hospital, Yancheng First Hospital Affiliated Hospital of Nanjing University Medical School, Yancheng, 224005, China.

**#: These authors contributed equally to this work.**

*** Corresponding author:**

Wen Bian, email: [bwycfy@163.com](mailto:bwycfy@163.com)

Lirong Huang, email: [ychlr009@163.com](mailto:ychlr009@163.com)

**Address:** Department of Cardiothoracic Surgery, Yancheng NO.1 People’s Hospital, Yancheng First Hospital Affiliated Hospital of Nanjing University Medical School, Yancheng, 224005, China.

**Supporting Experiments**

**1. Materials**

Methoxy poly(ethylene glycol) amine (MPEG-NH_2_, molecular weight (MW): 5000 Da) was obtained from JENKEM Technology Co., Ltd. (Beijing, China) and dehydrated by azeotrope with toluene. *N^ε^*-benzyloxycarbonyl-L-lysine-*N*-carboxyanhydride (Lys(Z)-NCA) was purchased from StruChem CO., LTD (Suzhou, China). Podophyllotoxin (PPT), paclitaxel (PTX), dry *N,N*-dimethylformamide (DMF), 2,3-dimethylmaleic anhydride (DMA), succinic anhydride (SA), and 3-mercaptopropionic acid were purchased from Aladdin Industrial Corporation (Shanghai, China). Cucurbitacin B was purchased from TCI Chemical Shanghai (Shanghai, China). ((3-(4,5-dimethylthiazol-2-yl)-2,5-diphenyl tetrazolium bromide (MTT), Reactive Oxygen Species Assay Kit, and DAPI were purchased from Beyotime Institute of Biotechnology (Shanghai, China).

**2. Characterization**

Gel permeation chromatography (GPC, Water E2695) was employed to evaluate the molecular weight and distribution (polydispersity index, PDI) of polymers. ^1^H nuclear magnetic resonance (^1^H NMR) spectrum was recorded on a Bruker AV 300 NMR system (Bruker Biospin, USA) at 300 MHz with deuterated methanol or deuterated dimethyl sulfoxide (DMSO-d6) as the solvent. The content of PPT and CuB were measured by high-performance liquid chromatography (HPLC) method on a Shimadzu HPLC system (LC-20A, Japan). For PPT detection, the detector was set at 292 nm using acetonitrile and water (80:20, v/v) as the mobile phase. For CuB detection, the detector was set at 230 nm using acetonitrile and water (40:60, v/v) as the mobile phase. Ultraviolet (UV) spectra were obtained using a UV-visible spectrophotometer (Shimadzu, UV-2450, Japan). Mass spectroscopy (MS) was performed using an AB SCIEX Triple Quad instrument (AB SCIEX, 5500, American). Size distribution and zeta potential were determined via dynamic light scattering (DLS, Zetasizer Nano Zs90; Malvern, England). The morphology of micelles were observed using transmission electron microscopy (TEM, JEM 2100, JEOL, Japan).

**3. Cell lines**

Human non-small cell lung cancer cell lines (A549 and drug-resistant A549/PTX) were supplied by KeyGEN BioTECH (Nanjing, China) and cultured in RPMI1640 containing 10% (v/v) fetal bovine serum, 100.0 IU/mL penicillin and 100.0 µg/mL streptomycin at 37°C in a humidified 5% CO_2_ atmosphere. Drug resistance of A549/PTX cells was maintained by the addition of Taxol (20.0 ng/mL) to the medium.

**4. Animals**

Kunming mice (male, 22.0-26.0 g, 5–6 weeks of age), and BALB/c nude mice (male, 18.0–20.0 g, 5–6 weeks of age) were purchased from the Vital River Laboratory Animal Technology Co., Ltd. (Beijing, China). All animals received care in compliance with the guidelines outlined in the Guide for the Care and Use of Laboratory Animals and the procedures were approved by the Wuhu NO.1 People’s Hospital.

**5. Synthesis of MPEG-*b*-PLL**

The block polymer methoxyl poly(ethylene glycol)-*block*-poly(L-lysine) (MPEG-*b*-PLL) was prepared in two steps. First, MPEG-NH_2_ was used as a macroinitiator to induce Lys(Z)-NCA ring-opening polymerization to obtain the methoxy polyethylene glycol-*block*-poly-ε-(benzyloxycarbonyl)-L-lysine (MPEG-*b*-PLLZ). Subsequently, the protection group, benzyloxycarbonyl, was removed to acquire the final polymer MPEG-*b*-PLL. Briefly, 2.0 g MPEG-NH_2_ was dissolved in 40.0 mL of DMF and was added to the Lys(Z)-NCA solution (5.0 g Lys(Z)-NCA dissolved in 30.0 mL dry DMF) via a syringe under a dry argon atmosphere. The mixture was stirred at 30°C for 72 h again under dry argon. At the end of the reaction, the mixture was precipitated with excess ice-cold diethyl ether to acquire the white product MPEG-*b*-PLLZ. The MPEG-*b*-PLLZ was dissolved in DMF and transferred into a dialysis bag (molecular weight cut-off, MWCO: 5000 Da) against DMF for 48 h to remove any unreacted Lys(Z)-NCA and MPEG-NH_2_. Finally, the DMF was removed under reduced pressure and dried under vacuum to obtain the purified MPEG-*b*-PLLZ (yield: 76.8%).

To deprotect the benzyloxycarbonyl groups of MPEG-*b*-PLLZ, 3.0 g of MPEG-*b*-PLLZ was dissolved in 30.0 mL of trifluoroacetic acid. Subsequently, 4.5 mL of 33% of HBr/acetic acid (wt%) was added to the MPEG-*b*-PLLZ solution at 0 °C and further stirred at 25°C for 40 min. Next, the mixture was precipitated by ice-cold diethyl ether to obtain a yellow precipitated product. The product was dissolved in dimethyl sulfoxide (DMSO) and dialyzed (MWCO: 5000 Da) against water for 48 h to remove any small molecular impurities, and the final product MPEG-*b*-PLL was obtained by lyophilization (white solid, yield: 71.3%).

**6. Synthesis of** **thioketal**

The thioketal (TK) linker was produced in accordance with a previous report.^[1]^ Typically, 3.0 g anhydrous 3-mercaptopropionic acid and 3.4 g anhydrous acetone was stirred under dry hydrogen chloride at room temperature for 6 h. Subsequently, the mixture was crystallized under an ice-salt mixture and filtered, washed with hexane and cold water each, for four times, and then vacuum dried to obtain a white crystal (yield: 86.5%).

**7. Protein adsorption**

Protein adsorption was evaluated using bovine serum albumin (BSA) as the model protein in accordance with a previous report.^[2]^ Briefly, 1.0 mL of micelle solution (2 mg/mL) was incubated with 1.0 mL of BSA solution (4 mg/mL) at pH 7.4 and 6.8 at 37°C for 12 h, respectively. At the end of the incubation, 1.0 mL of each sample was withdrawn centrifuged at 8000×g for 10 min to precipitate the protein-adsorbed micelles. The amount of protein in the supernatant was measured by UV-visible spectroscopy at 280 nm, and the amount of BSA adsorbed on the micelles was then calculated.

**8. Critical micelle concentration detection**

The polymers were dissolved in PBS at various concentrations. Subsequently, the Nile red solution (1.0 mg/mL in DMSO) was added to the final concentration of 6.0 × 10^-7^ mmol/L. Fluorescent spectroscopy (F-7100, Hitachi, Japan) was used to determine the fluorescence emitted by the solution (*λ*ex = 557 nm, *λ*ex = 601 nm). The intersection of the fluorescence intensity of the highest and lowest concentrations was determined as the critical micelle concentration (CMC) value.

**9. Stability analysis**

Micelles were incubated in PBS and RPMI1640 contain 10% fetal bovine serum (FBS), respectively. At designed time intervals, the diameters of micelles were tested by DLS.

**Supporting Figures and Tables**





**Fig. S1** The synthesis route of MPEG-*b*-PLL-TKPPT/DMA.


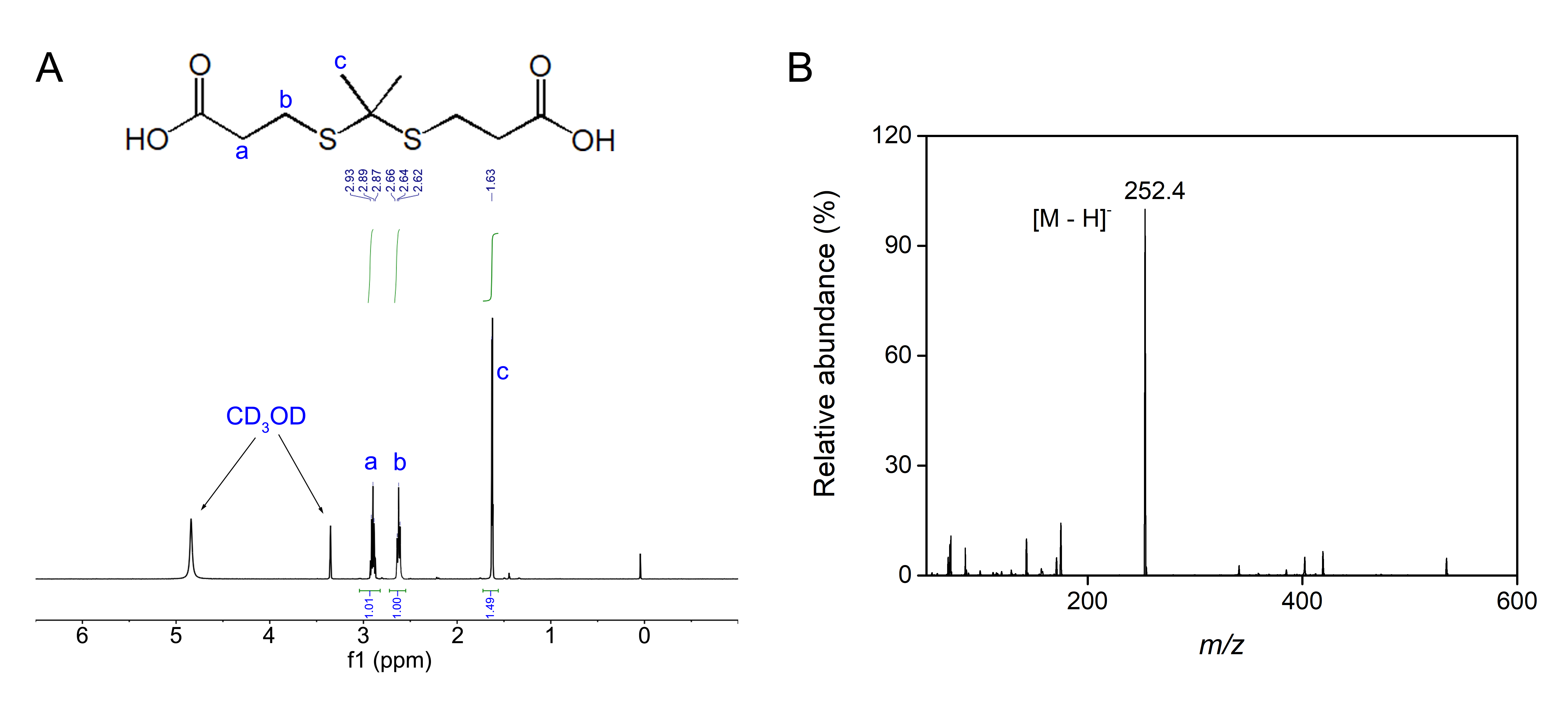


**Fig. S2** Characterization of TK. (A) ^1^H NMR spectrum of TK; (B) MS spectrum of TK.


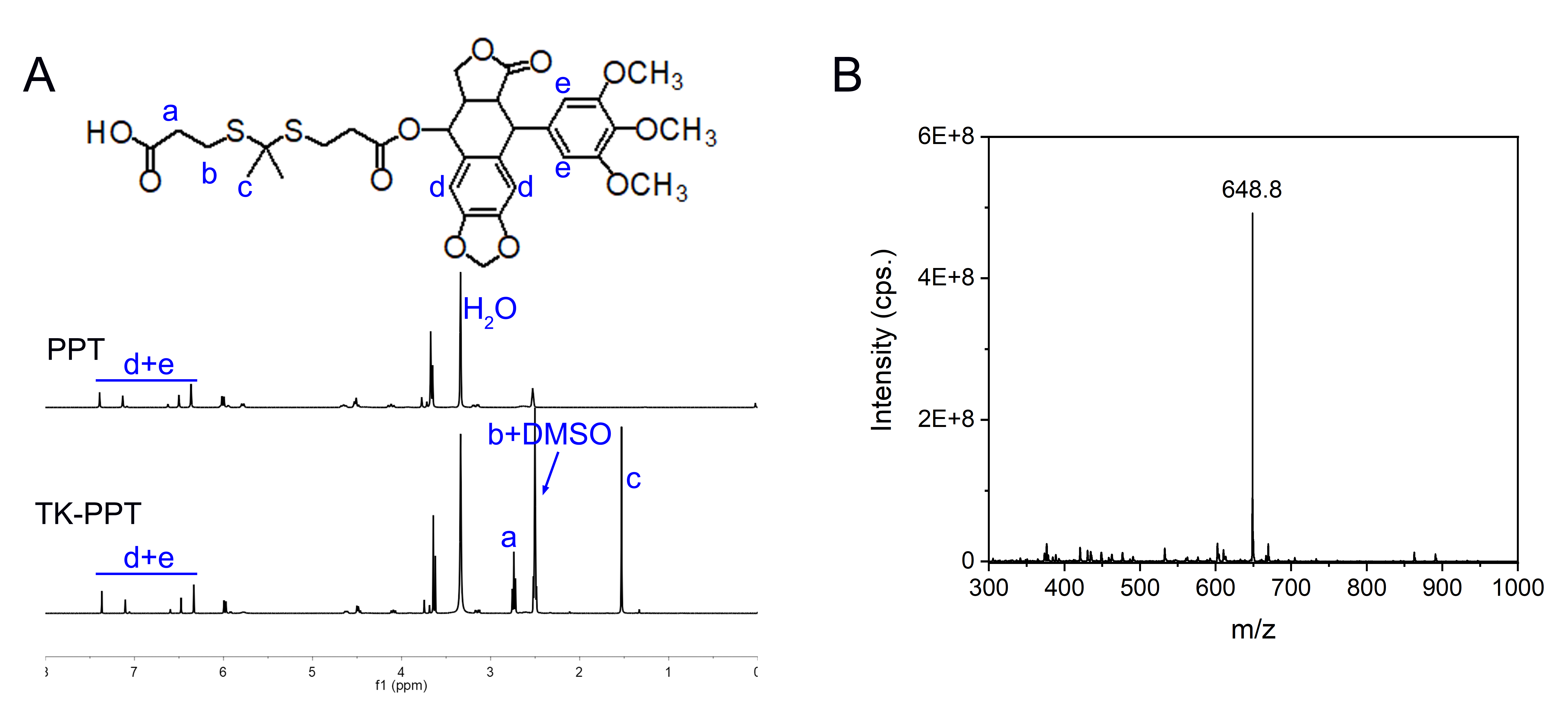


**Fig. S3** Characterization of TK-PPT. (A) ^1^H NMR spectrum of PPT and TK-PPT; (B) MS spectrum of TK-PPT.


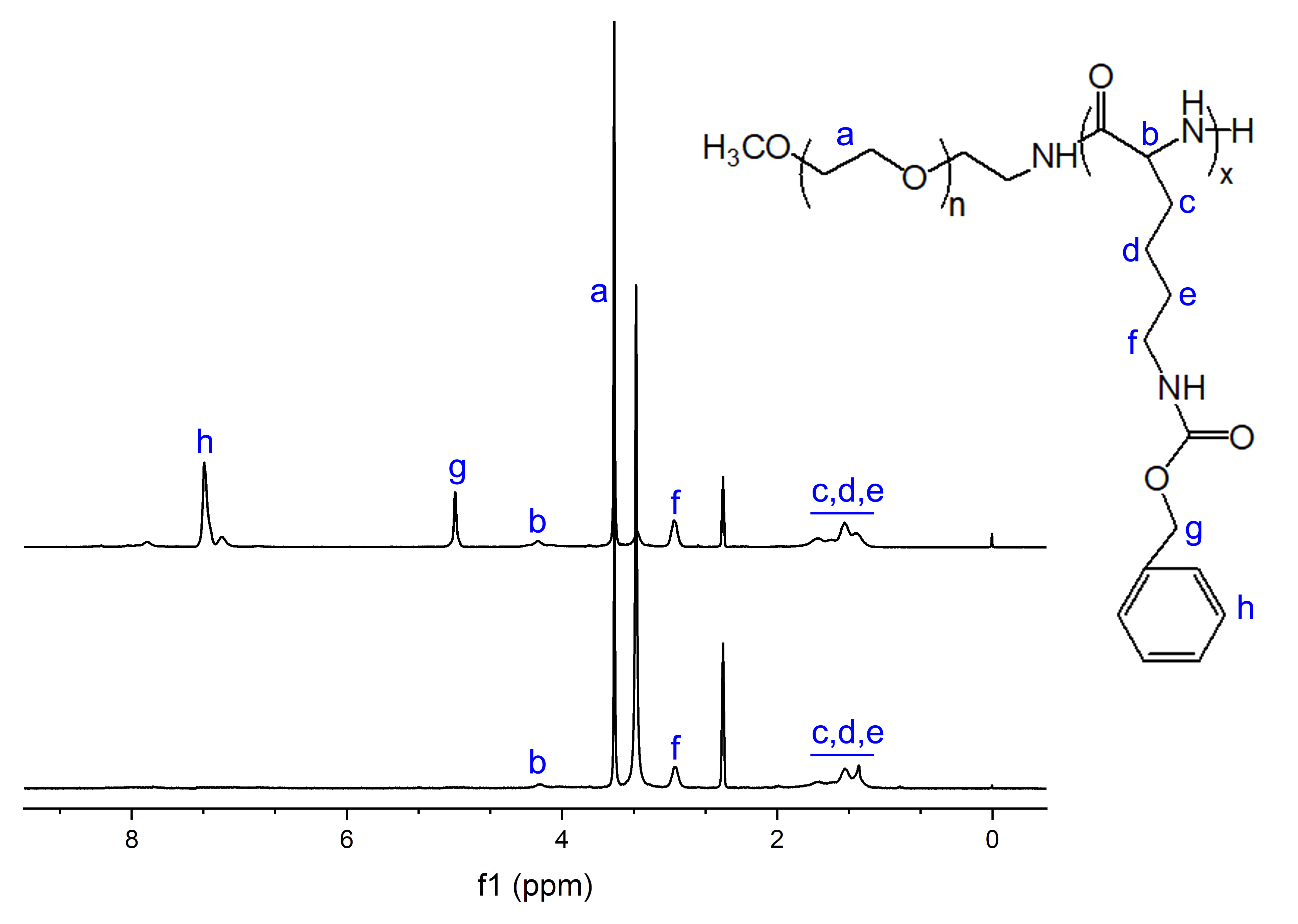


**Fig. S4** ^1^H NMR spectrums of MPEG-*b*-PLLZ and MPEG-*b*-PLL in DMSO-*d6*.


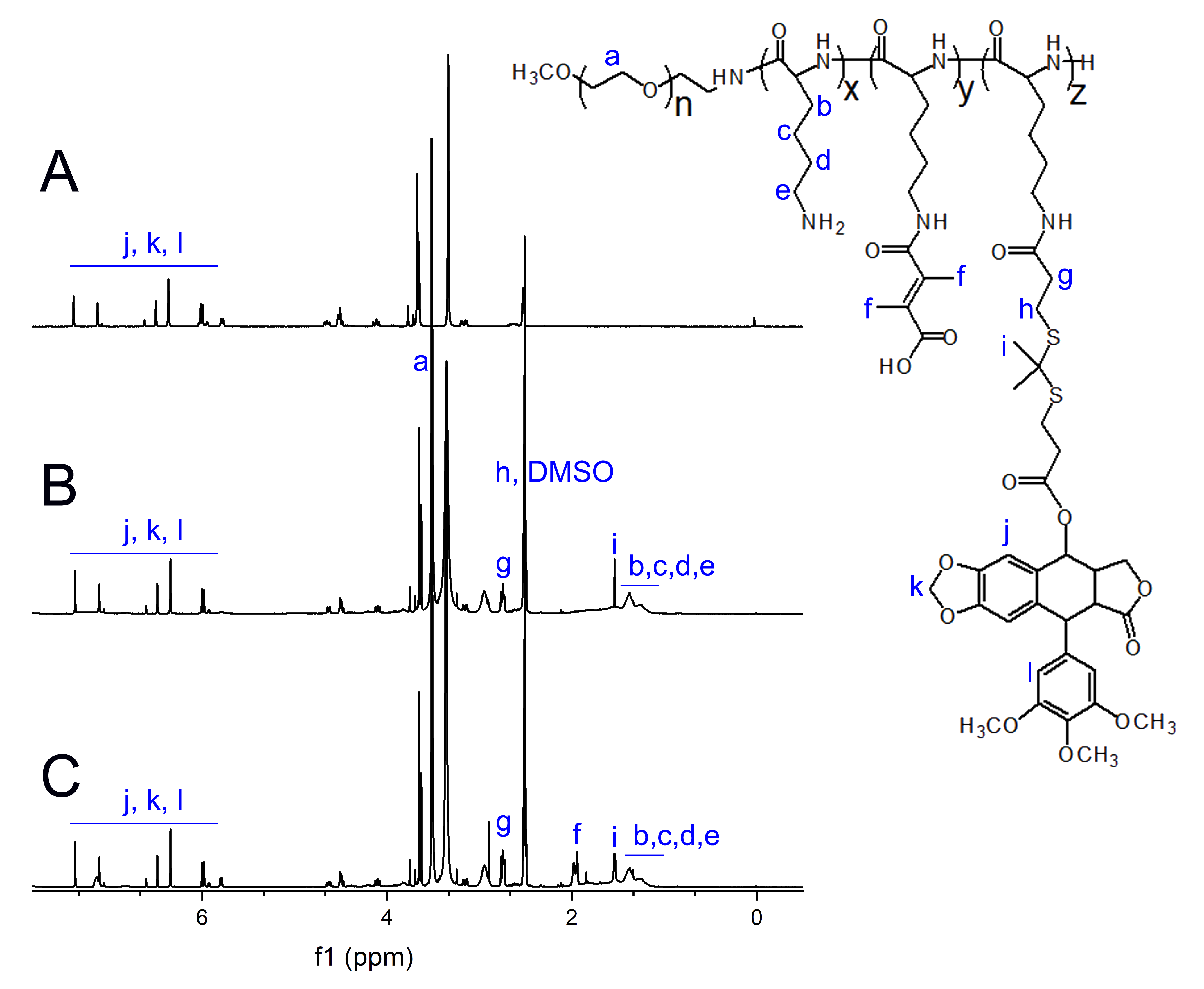


**Fig. S5** ^1^H NMR spectrums of PPT (A), MPEG-*b*-PLL-TKPPT (B), and MPEG-*b*-PLL-TKPPT/DMA (C) using DMSO-*d6* as the solvent.


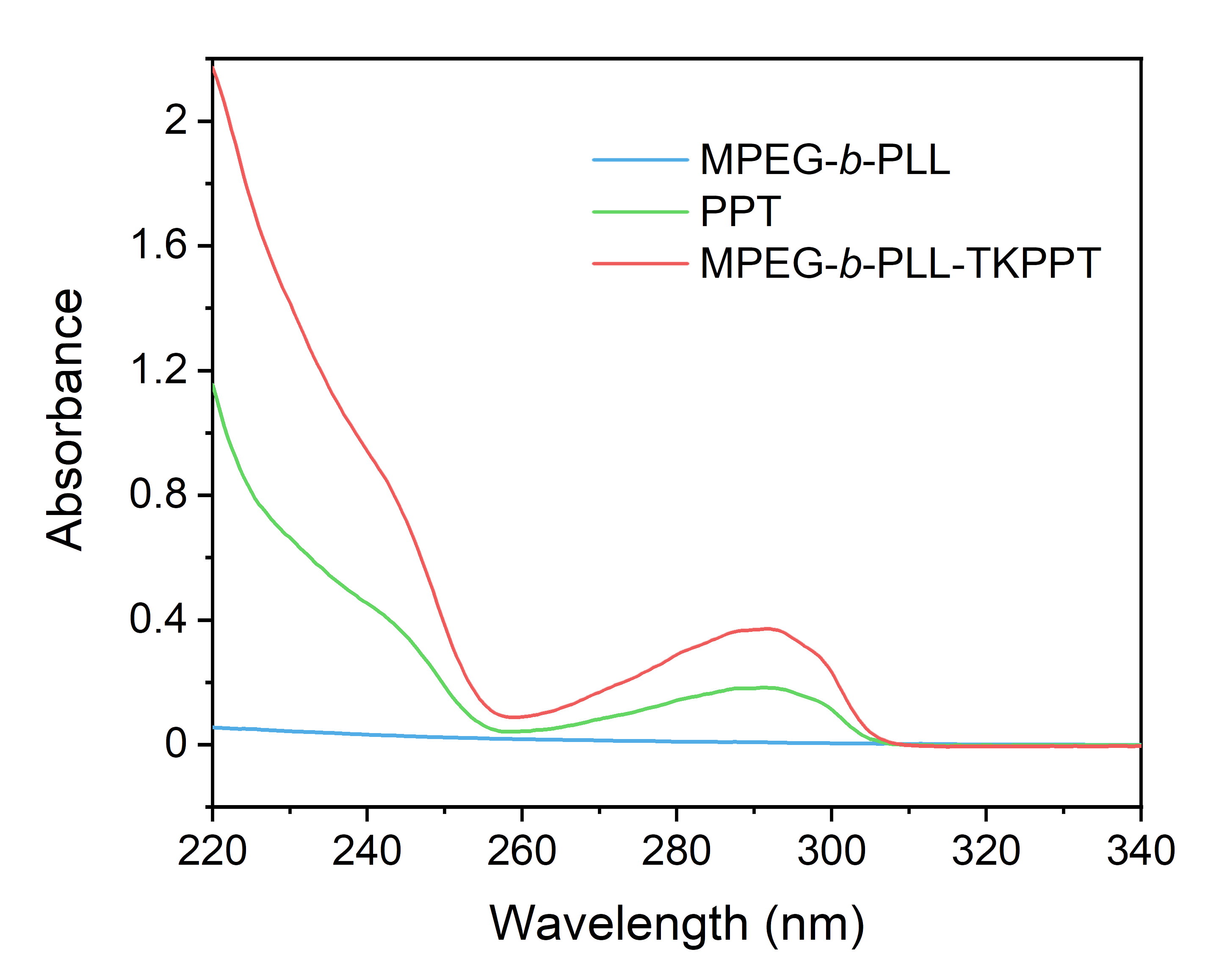


**Fig. S6** UV spectrums of MPEG-*b*-PLL, PPT, and MPEG-*b*-PLL-TKPP in DMSO.


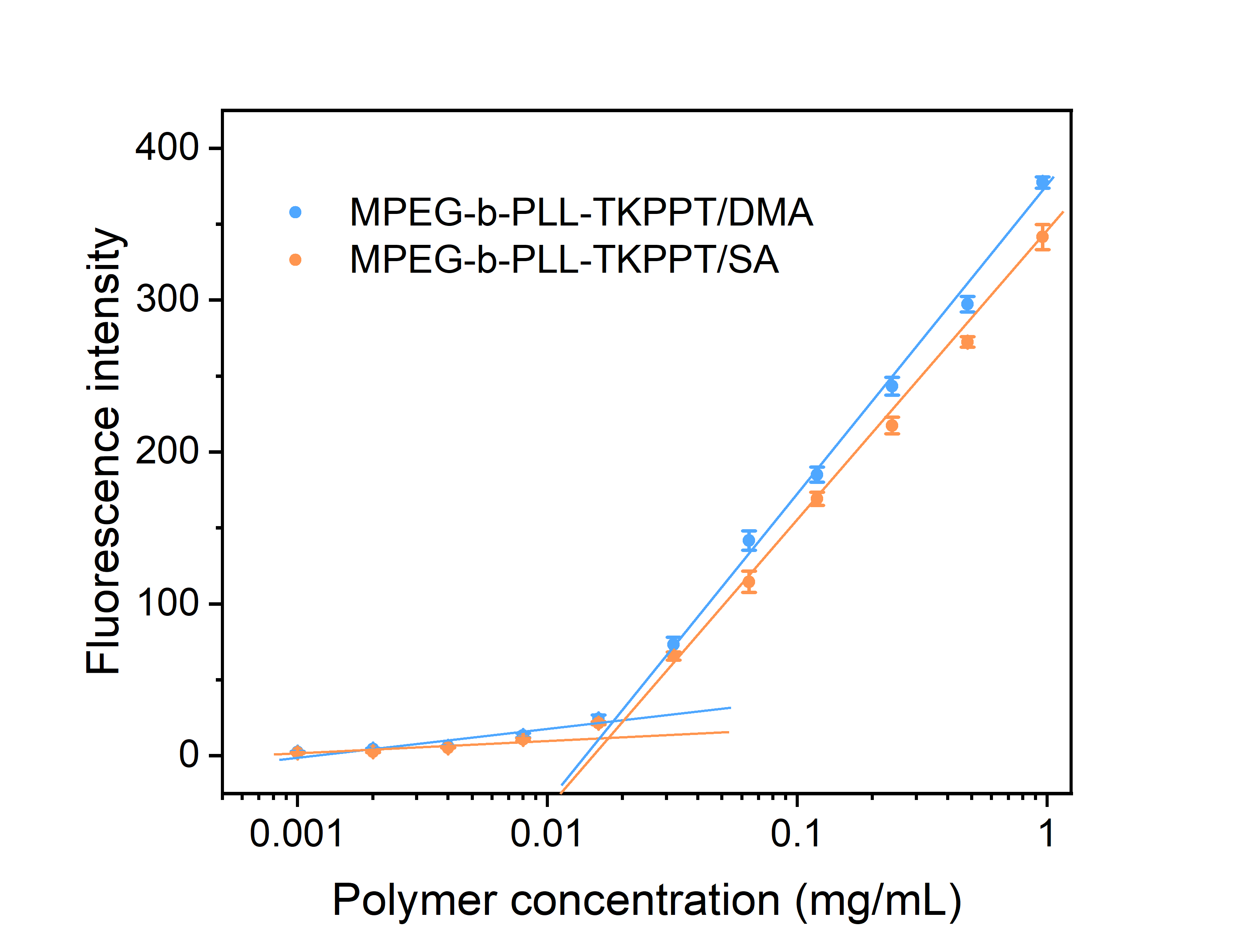


**Fig. S7** CMC detection of MPEG-*b*-PLL-TKPP/DMA and MPEG-*b*-PLL-TKPP/SA.


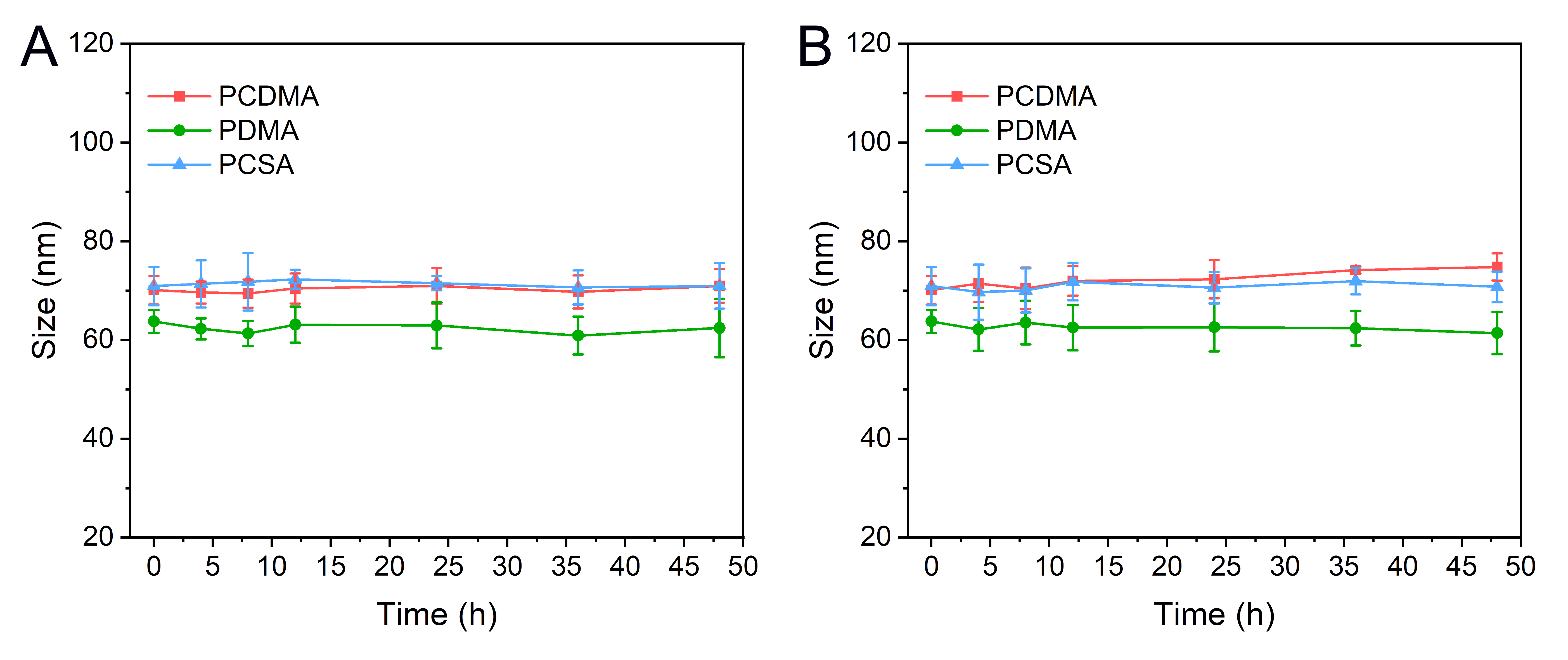


**Fig. S8** Size changes of PCDMA, PDMA, and PCSA in PBS (A) and RPMI 1640 with 10% FBS (B).





**Fig. S9** Mechanism for hydrolysis of 2,3-dimethylmaleic amide at pH 6.8.

| **Table S1.** Characterization of polymers | | | | |
| --- | --- | --- | --- | --- |
| Polymer | Composition ratio^a,b^ | *M_w_*^a^ (Da) | *M*_w_^b^ (Da) | PDI^b^ |
| MPEG-*b*-PLLZ | 113: 30 | 13440 | 13897 | 1.15 |
| MPEG-*b*-PLL | 113: 30 | 8480 | 8532 | 1.09 |
| MPEG-*b*-PPL-TKPPT | 113: (20: 10) | 14730 | 15327 | 1.18 |
| MPEG-*b*-PPL-TKPPT/DMA | 113: (3: 10: 17) | 16566 | 16973 | 1.21 |
| MPEG-*b*-PLL-TKPPT/SA | 113: (4: 10: 16) | 16042 | 16224 | 1.16 |
| ^a^ Estimated by ^1^H NMR  ^b^ Detected by GPC  PDI: polydisperse index | | | | |

| **Table S2.** Components of micelles. | | | |
| --- | --- | --- | --- |
| Micelles | MPEG-*b*-PLL-TKPPT/DMA | MPEG-*b*-PLL-TKPPT/SA | CuB |
| PDMA | + | - | - |
| PCDMA | + | - | + |
| PCSA | - | + | + |
| “-” means no this part; “+” means have this part. | | | |

| **Table S3.** Main properties of micelles. | | | | | | | |
| --- | --- | --- | --- | --- | --- | --- | --- |
| Micelles | Size^a^ (nm) | Zeta potential^a^ (mV) | PDI^a^ | DLE (%) | | DEE (%) | |
|  |  |  |  | PPT | CuB | PPT | CuB |
| PCDMA | 70±3 | -16.5±1.8 | 0.208±0.027 | 19.4±1.2 | 4.7±0.6 | - | 56.1±3.4 |
| PDMA | 64±2 | -17.1±0.6 | 0.229±0.024 | 23.4±1.3 | - | - | - |
| PCSA | 71±4 | -19.5±0.9 | 0.206±0.018 | 20.4±1.4 | 5.0±0.5 | - | 54.8±2.6 |
| ^a^: Detected by dynamic light scattering; PDI: Polydispersity index; DLE: Drug loading efficiency. | | | | | | | |

| **Table S4.** The IC50 values and resistance index (RI) of PTX, PPT, and PCDMA on A549 and A549/PTX cells for 48 h (unit of IC50 value: μg/mL). | | | |
| --- | --- | --- | --- |
|  | A549/PTX | A549 | RI |
| PTX | 5.71 | 0.04 | 142.8 |
| PPT | 0.77 | 0.38 | 2.0 |
| PCDMA | 10.97 | 7.72 | 1.4 |

**References**

[1] Hu J-J, Lei Q, Peng M-Y, et al. A positive feedback strategy for enhanced chemotherapy based on ROS-triggered self-accelerating drug release nanosystem. *Biomaterials* 2017;128:136-46.

[2] Dai L, Li X, Duan X, et al. A pH/ROS Cascade-Responsive Charge-Reversal Nanosystem with Self-Amplified Drug Release for Synergistic Oxidation-Chemotherapy. *Advanced Science* 2019;6:1801807.
